# Supplementary material for: Systematic analysis of alternative splicing in time course data using Spycone
Source: Bioinformatics. 2022 Dec 29;39(1):btac846. doi: 10.1093/bioinformatics/btac846 (PMC9831059; doi:10.1093/bioinformatics/btac846)
Supplement: btac846_Supplementary_Data [file btac846_supplementary_data.zip › spycone-supplementary-figures.pdf]

Supplementary figures

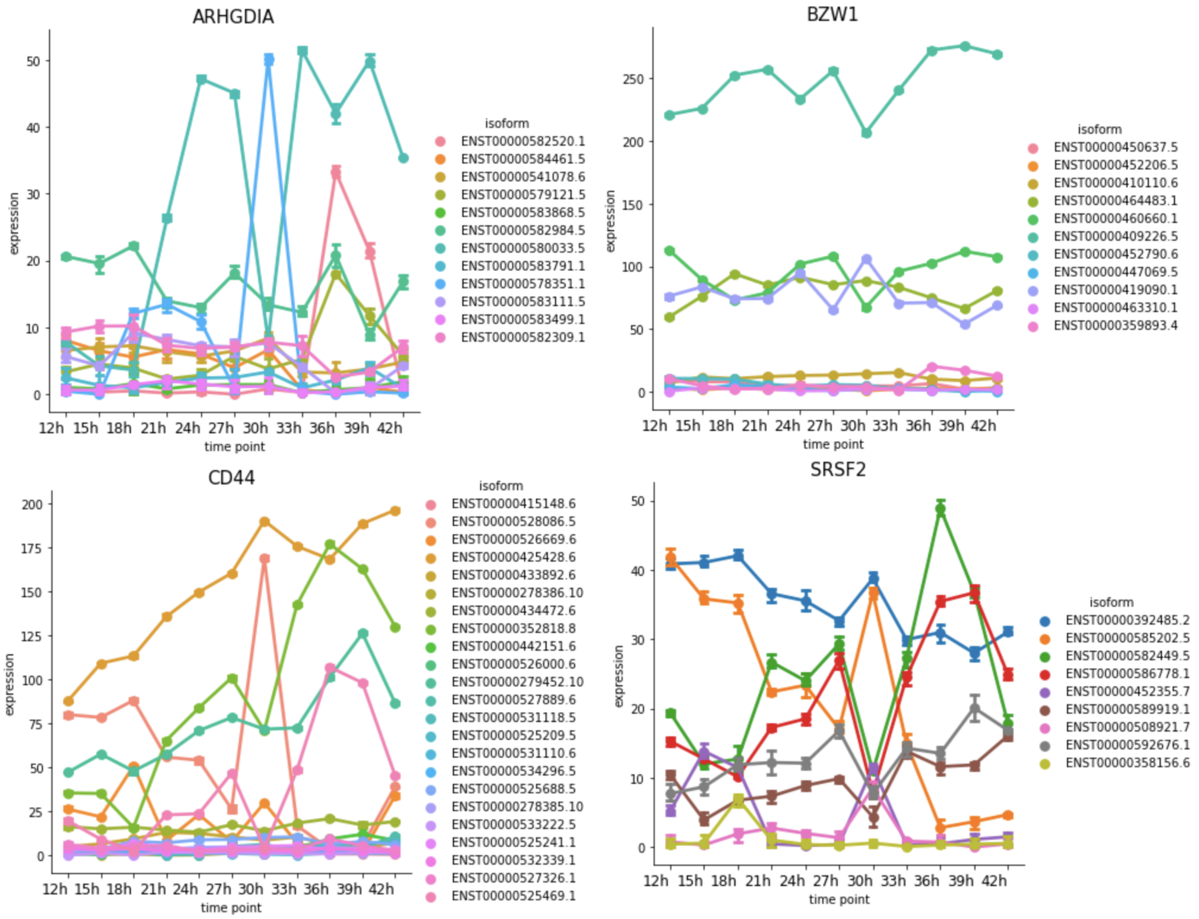

S1. Real life IS examples that are challenging to detect. In the case of gene ARHGDIA, at 30h a lowly expressed isoform (light blue) is up-regulated while the highest abundance isoform. In the case of CD44, a lowly expressed isoform (pink) is up-regulated at 30h, however, there is no other isoform down-regulated with the similar magnitude as in ARHGDIA. Similar case in SRSF2, with an isoform (orange) up-regulated at 30h, multiple isoforms down-regulated at the same time point. This case could be an example of multiple isoform switching. In BZW1 gene, two isoforms seem to switch at 30h and 33h, however, in the earlier time points, the two isoforms are expressed in parallel.

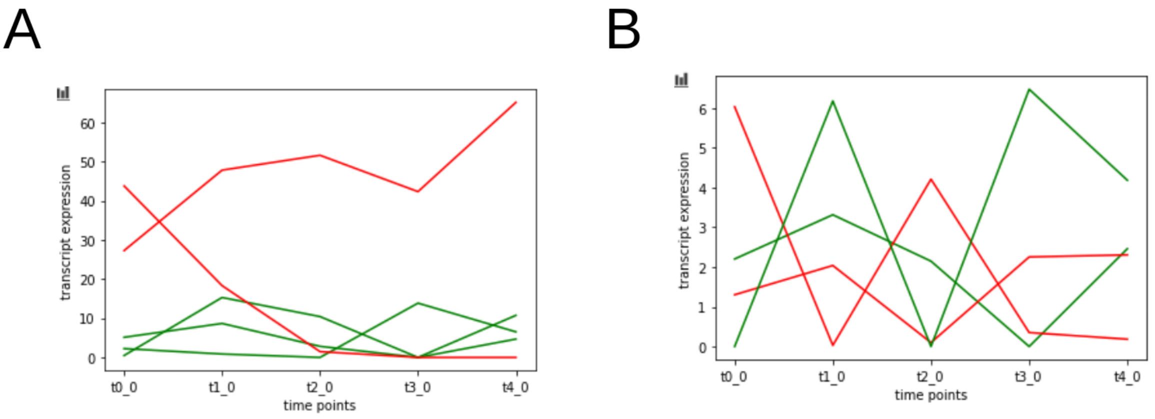

S2. Model 1 and Model 2 examples from simulated data. Model 1 allows switching only with the highest abundance isoform. Model 2 allows isoforms with higher than 0.3 abundance to switch.

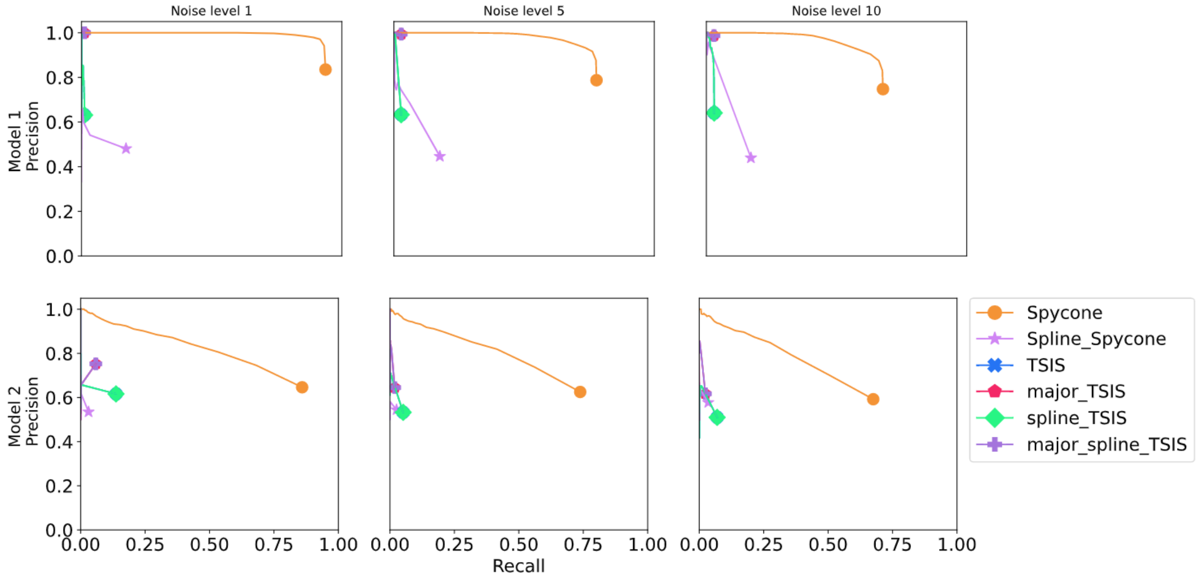

S3. Spline regression model with simulated dataset generated by the two models. The spline regression is applied to detect switching points. The resulting switch points are then tested for their significance using Spycone and TSIS and calculated the precision and recall as described in the manuscript.

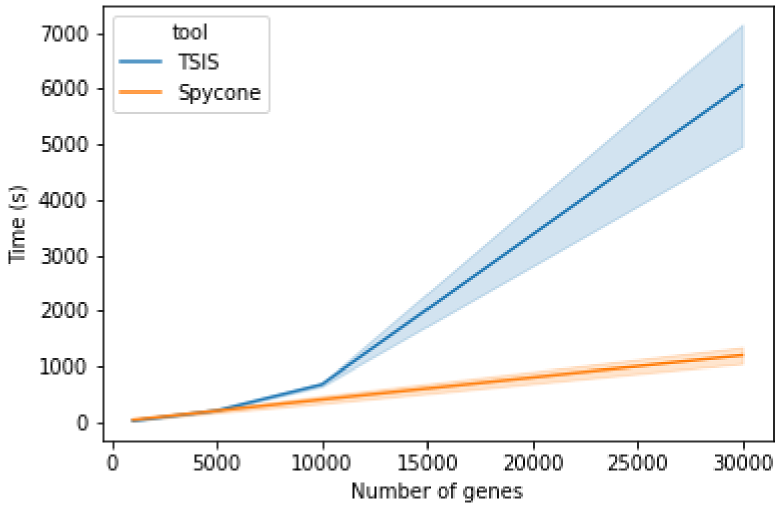

S4. Computation time for Spycone and TSIS (seconds) on simulated datasets ranging from 1000 to 30000 genes with 3 to 4 replicates. Tested on a laptop device with Intel CoreTM i7-10510U CPU @ 1.8GHz x 8 cores, 16GB RAM, 512GB disk capacity.

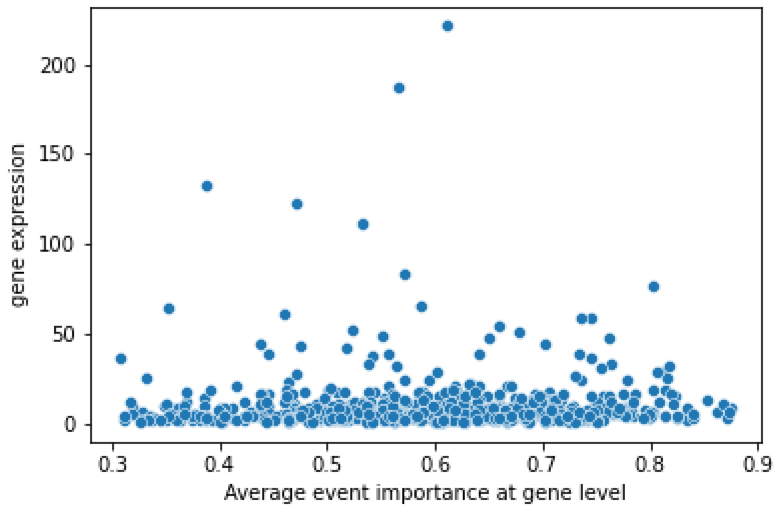

S5. Relationship between event importance and overall gene expression from Spycone results.

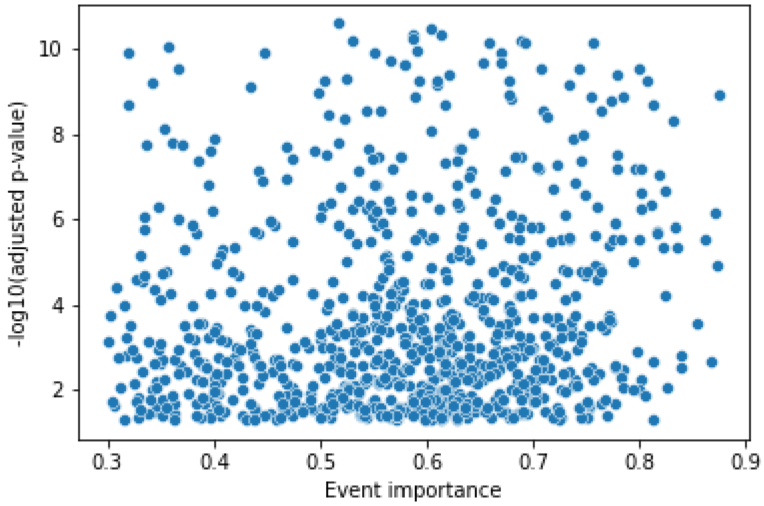

S6. Relationship between event importance and adjusted p-value from Spycone results.

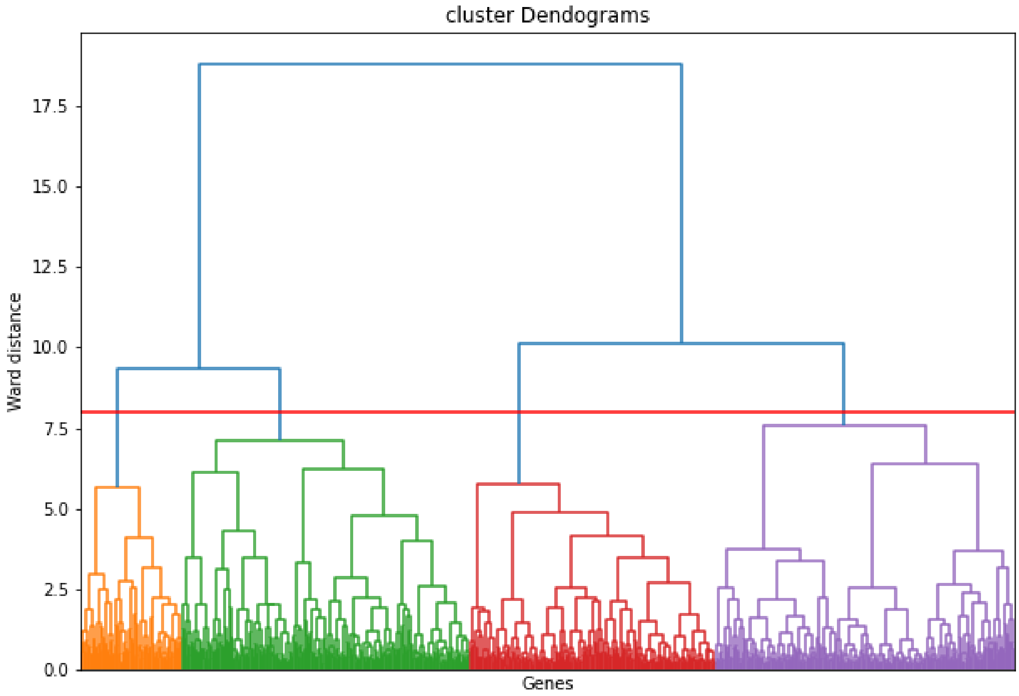

S7. Cluster dendrogram of hierarchical clustering SARS-Cov-2 dataset. Each cluster is colored with different colors under the ward distance threshold at 8.

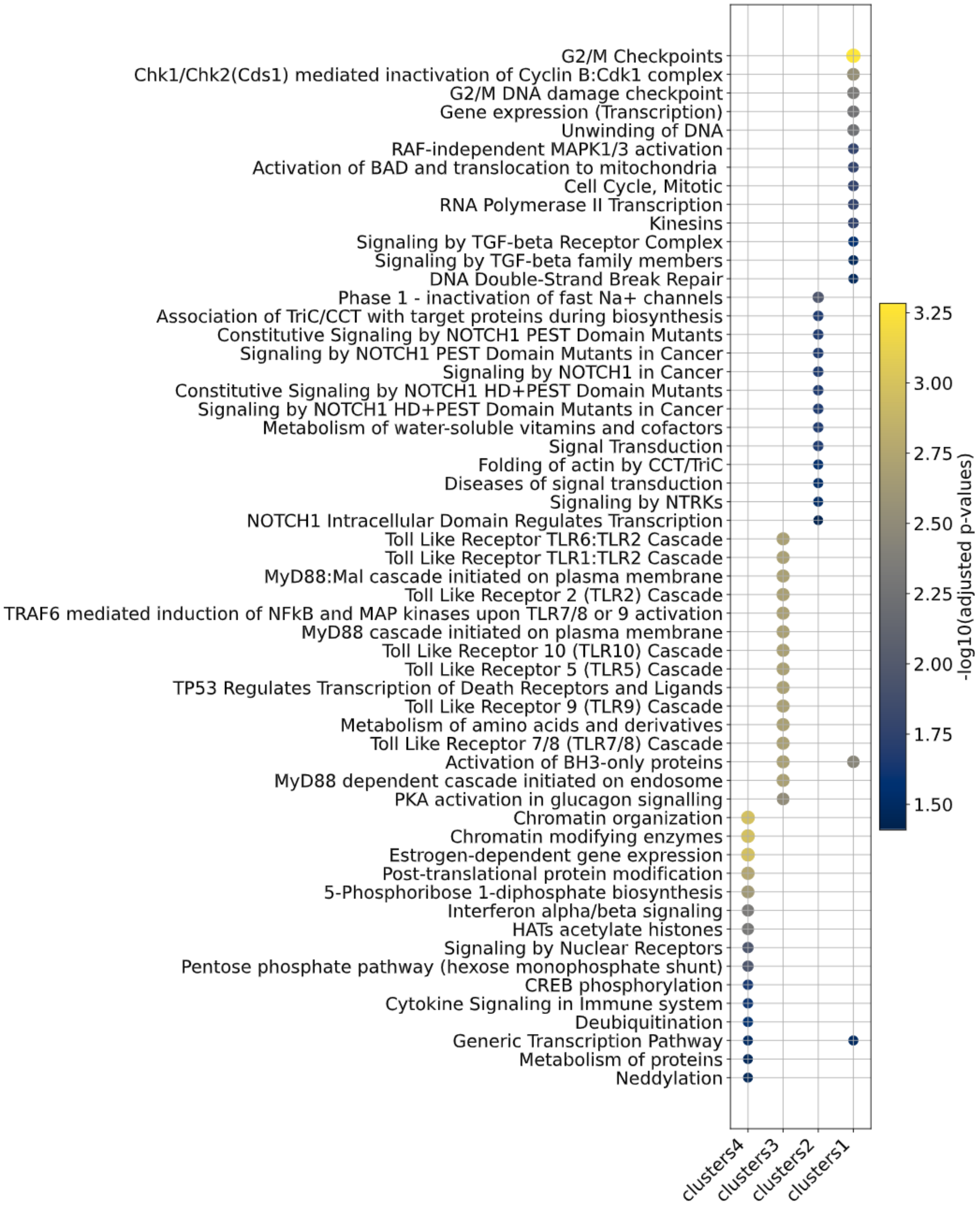

S8. NEASE enrichment results with Reactome pathways (y-axis) of the four clusters from Spycone (x-axis). The dotplot shows the -log10(adjusted p-value) from the hypergeometric test.

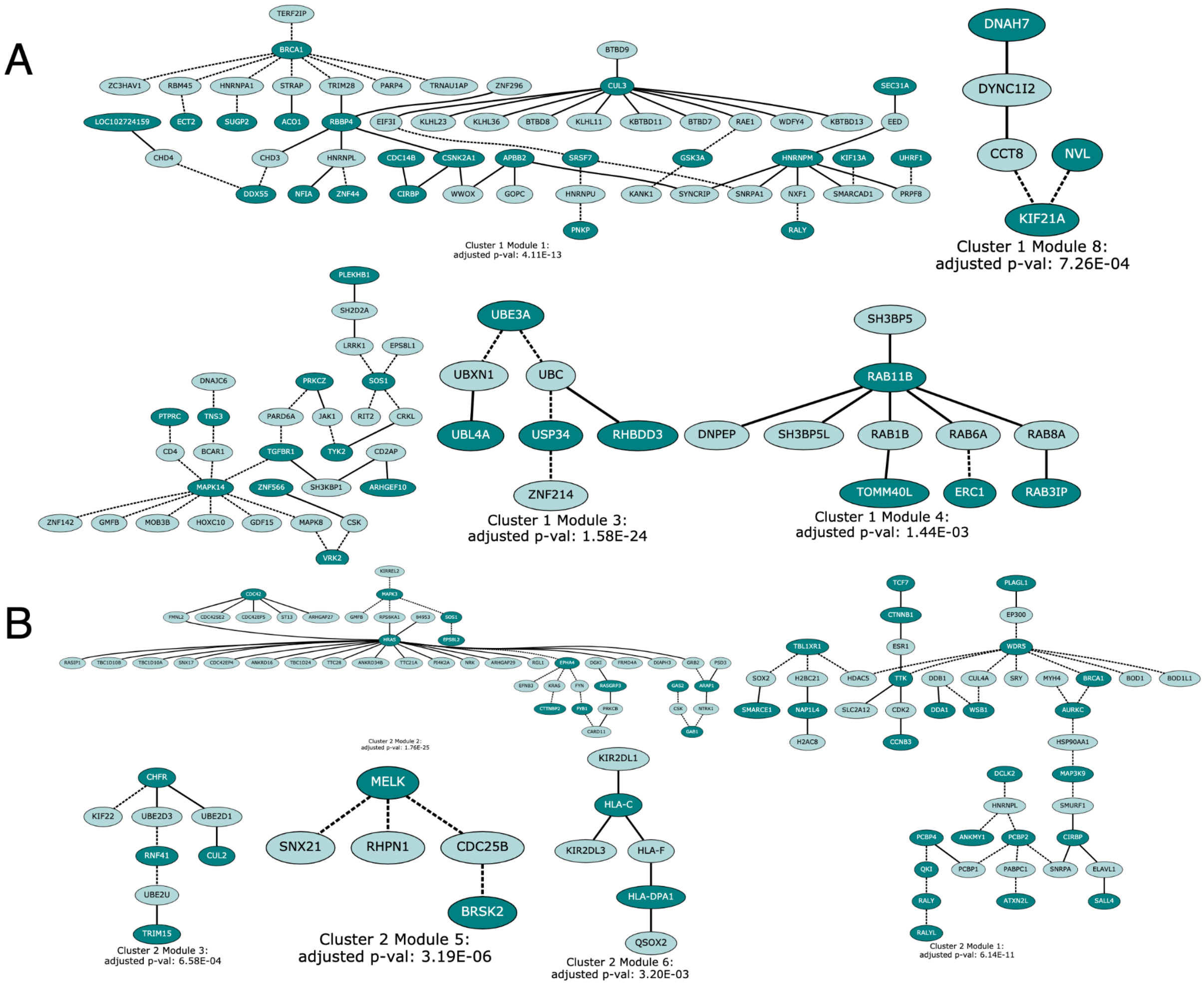

S9. Spycone results in modules of PPI network and their gene set enrichment results. Active network modules are identified using DOMINO. Each node represents a domain of a gene. Purple nodes are the isoform switched genes and orange nodes are non-IS genes from the PPI. Dashed edges are the affected interactions between the genes due to the lost/gained of domains during the IS events.

C

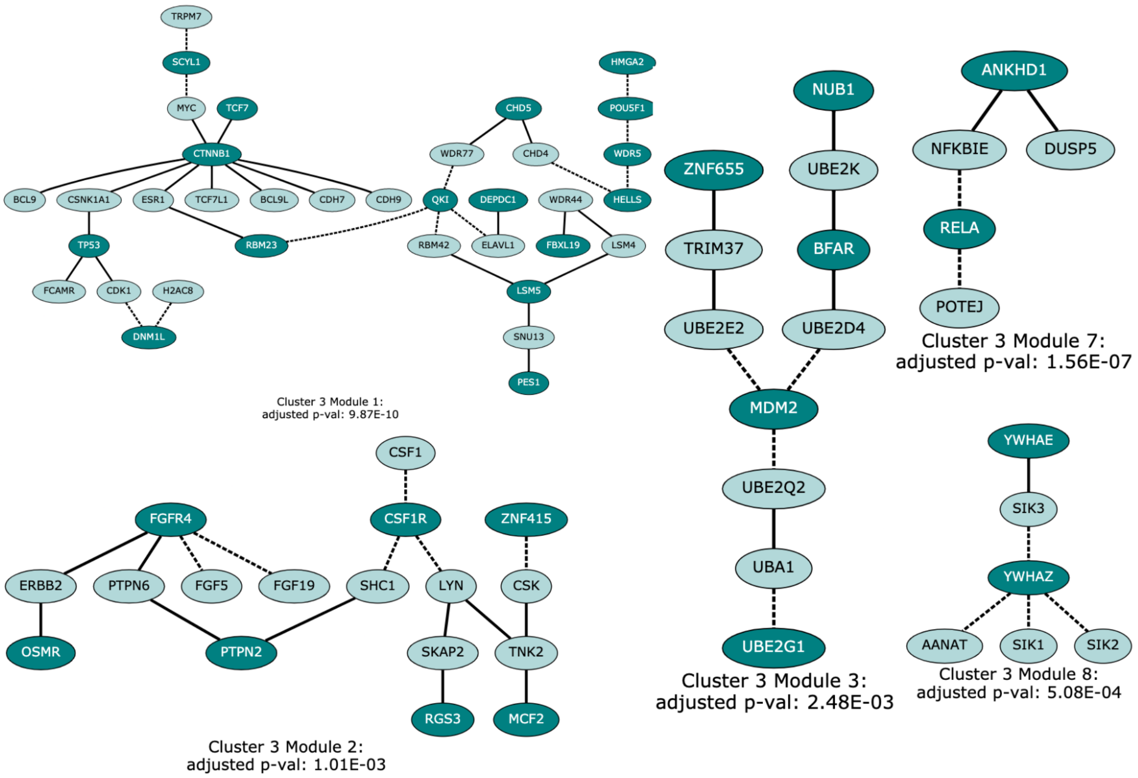

D

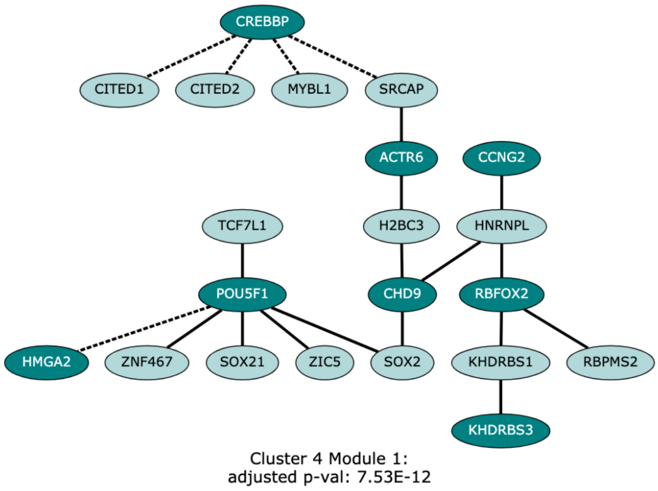

S9. (Cont). Spycone results in modules of PPI network and their gene set enrichment results. Active network modules are identified using DOMINO. Each node represents a domain of a gene. Purple nodes are the isoform switched genes and orange nodes are non-IS genes from the PPI. Dashed edges are the affected interactions between the genes due to the lost/gained of domains during the IS events.

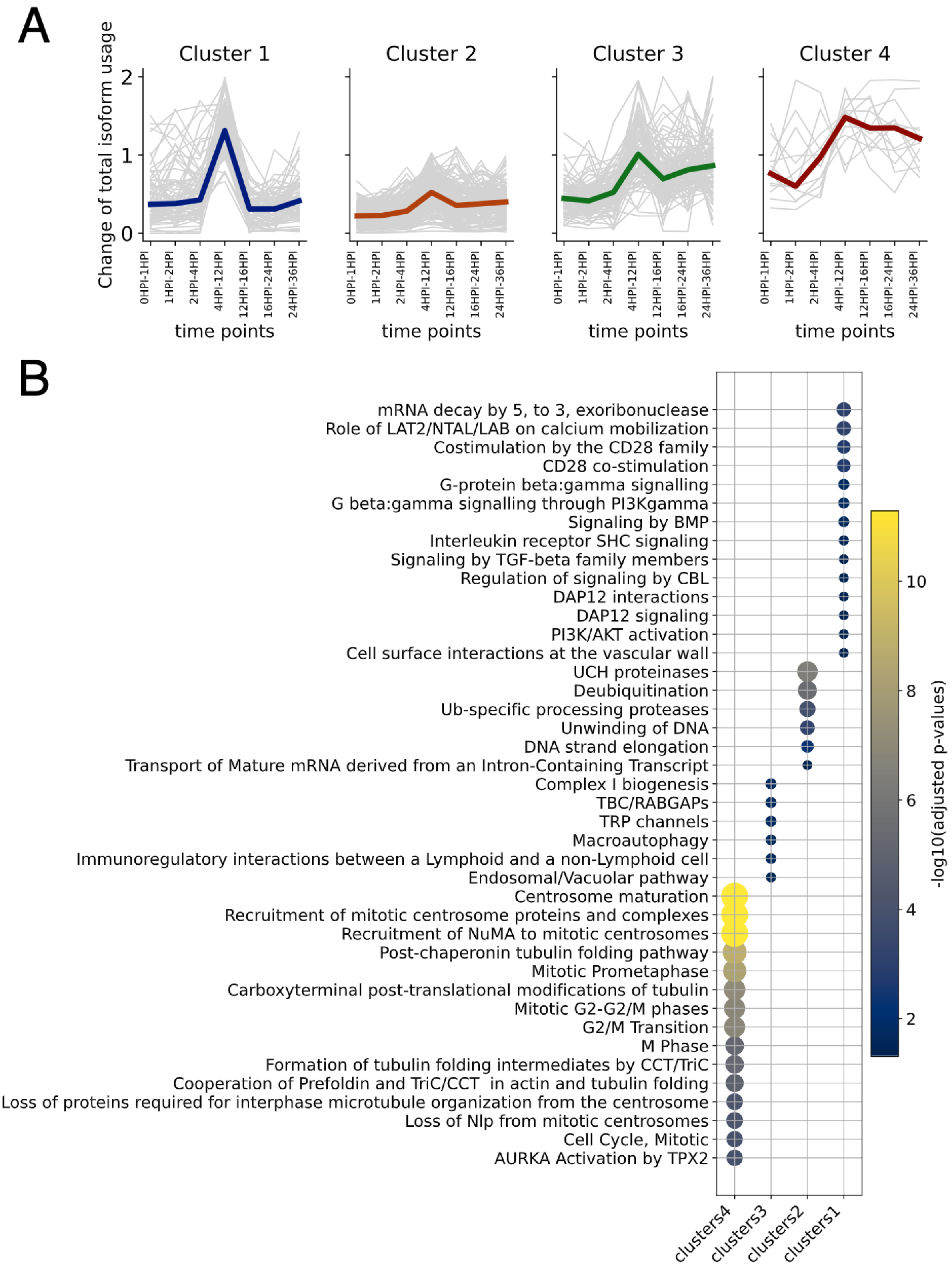

S10. Clustering and NEASE results from TSIS identified IS genes. Overall, clusters with similar prototypes from both tools are enriched in distinct pathway terms. For example, TSIS's cluster 1 and Spycone's cluster 1 have a strong peak between 4 and 12 hours post infection. Only TGF-beta signaling is commonly found in both tools. MAPK pathway and DNA damage checkpoint are enriched uniquely in Spycone. TSIS's Cluster 2 and Spycone's Cluster 3 have lower changes of total isoform usage overall. Spycone's clusters showed more unique and relevant terms: 70 enriched Reactome terms in Spycone's clusters and only 7 terms in TSIS's clusters. TSIS's cluster 3 and Spycone cluster

2 show an increase of change of total isoform usage after 12 hours post infection. Spycone's cluster is enriched uniquely in protein folding chaperonin complex TriC/CCT and NOTCH signaling pathway. Finally, TSIS's cluster 4 and Spycone's cluster 4 have increasing changes of total isoform usage overall. TSIS's cluster is enriched in mitosis-related pathways, cell cycle, and tubulin folding. Whereas in Spycone's cluster 4 is found with signaling by PTK6, interferon, metabolism of proteins, pentose phosphate pathway etc.
